# Tongjiang Hewei Decoction Improves Airway Hyperresponsiveness in Gastroesophageal Reflux Cough by Inhibiting ADAM33 and Epac1/Rap1 Pathway

**DOI:** 10.1002/fsn3.71223

**Published:** 2025-12-18

**Authors:** Xiulian Zhang, Xueliang Li, Yanmei Cheng, Lei Wei, Fangying Liu, Li Li, Wei Zhang, Xiuli Yan

**Affiliations:** ^1^ Baoshan Hospital of Shanghai University of Traditional Chinese Medicine Shanghai China; ^2^ Baoshan Branch, Ren Ji Hospital, School of Medicine, Shanghai Jiaotong University Shanghai China; ^3^ Shanghai University of Traditional Chinese Medicine Shanghai China; ^4^ Shuguang Hospital of Shanghai University of Traditional Chinese Medicine Shanghai China; ^5^ Yueyang Hospital of Integrated Traditional Chinese and Western Medicine, Shanghai University of Traditional Chinese Medicine Shanghai China

**Keywords:** ADAM33, airway hyperresponsiveness, Epac1/Rap1 signaling pathway, gastroesophageal reflux cough, Tongjiang Hewei Decoction

## Abstract

Gastroesophageal reflux disease is a common condition that can lead to various complications, with gastroesophageal reflux cough (GERC) being one of the notable manifestations. Despite conventional therapies targeting acid suppression, recurrence and incomplete efficacy remain significant challenges. Tongjiang Hewei Decoction (THD), a traditional Chinese formulation, has shown clinical promise in alleviating GERD‐related symptoms. However, its therapeutic mechanisms in GERC remain unexplored. In this study, the airway hyperresponsiveness (AHR) guinea pig model was constructed by infusing hydrochloric acid into the lower esophagus. Transcriptome sequencing was used to identify THD‐related possible pathways in GERC. Lung resistance was measured to evaluate lung function. Hematoxylin–eosin staining, immunohistochemistry, real‐time fluorescence quantitative polymerase chain reaction, and western blotting were employed to assess airway remodeling. Airway smooth muscle cells were isolated to further investigate the effects of THD in vitro. In vivo assay indicated that THD reduced lung resistance and attenuated bronchial wall thickening, mucus hypersecretion, and inflammatory infiltration in a dose‐dependent manner. Mechanistically, THD suppressed ADAM33, RhoA/ROCK, and Epac1/Rap1 pathways in vivo, correlating with reduced α‐SMA and sm‐MHC expression. Transcriptomic analysis revealed that THD exerted its effects through the Epac1/Rap1 signaling pathway. The Rap1 activator reversed THD's anti‐AHR effects. In vitro, THD inhibited contractile protein synthesis via ADAM33 silencing, while ADAM33 overexpression abolished this effect. Collectively, THD alleviated GERC‐induced AHR through dual modulation of the ADAM33/RhoA/ROCK axis and Epac1/Rap1 signaling, providing novel mechanistic insights into its therapeutic potential. These findings position THD as a multifaceted candidate for GERC management, bridging traditional medicine with modern molecular pharmacology.

## Graphical abstract

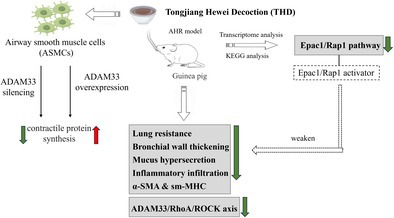

## 1 Introduction

Gastroesophageal reflux disease (GERD) is a common condition that can lead to various complications, with gastroesophageal reflux cough (GERC) being one of the notable manifestations (Dunbar 2024; Sharma and Yadlapati 2021). The pathogenesis involves microaspiration of gastric acid directly irritating the respiratory mucosa, esophago‐bronchial reflex activation via vagal pathways, and airway hyperresponsiveness (AHR) mediated by chronic inflammation and neural remodeling (Sharma and Yadlapati 2021; Amarasiri et al. 2013). The acid reflux can irritate the esophagus lining, causing inflammation and discomfort, which often culminates in GERC (Zhang et al. 2023). Clinically, GERC imposes a substantial burden on quality of life, triggering asthma attacks (Mallah et al. 2022). Current treatment for GERD and GERC primarily focuses on lifestyle modifications, pharmacotherapy, and, in refractory cases, surgical interventions (Katzka and Kahrilas 2020). Pharmacological treatments often involve antacids, H2 blockers, and proton pump inhibitors (PPIs), which aim to reduce the production of stomach acid (Maret‐Ouda et al. 2020). However, these treatments have limitations, including potential side effects, recurrence after cessation of medication, and unsuitability for all patients. Hence, it is necessary to pursue more efficacious treatment modalities for GERC.

In recent years, there has been growing interest in the use of traditional Chinese medicine (TCM) as an alternative or complementary approach to managing GERD and GERC (Dai et al. 2020; Song et al. 2024). TCM emphasizes holistic regulation of gastrointestinal motility, inflammation, and neural sensitization (Peng et al. 2022). The reformulated Xiao Chai Hu Tang exhibits a therapeutic efficacy akin to that of omeprazole in individuals grappling with mild to moderate GERD, as it bolsters the pressure of the lower esophageal sphincter and curtails ineffective swallowing (Li et al. 2021). Li et al. discern that Sini Zuojin Decoction exerts a conspicuously beneficial impact on GERD by alleviating symptoms such as heartburn, acid regurgitation, and reflux (Li et al. 2020). Likewise, Banxia Hou Po Decoction proves efficacious in the treatment of GERD (Song et al. 2025). Tongjiang Hewei Decoction (THD) is a TCM prescription developed for GERC, which is characterized by regulating qi and descending adverse qi, clearing heat and resolving phlegm, offering a promising alternative and garnering attention for its efficacy in managing GERD symptoms (Liu CF et al. 2013). Clinical studies suggest that Tongjiang Hewei Fang not only alleviates symptoms such as heartburn and regurgitation but also improves overall quality of life, with the potential to reduce symptom recurrence rates (Li et al. 2022). Yang et al. unveil that Tong Jiang Mixture possesses the capability to impede the PI3K/AKT pathway and suppress NF‐κB, thereby attenuating inflammatory responses and ameliorating reflux esophagitis (Lu et al. 2025). Nevertheless, the exact mechanisms underlying the therapeutic efficacy of THD in the treatment of GERC remain to be further elucidated.

The ADAM33 gene encodes a protein that is a member of a disintegrin and metalloproteinase (ADAM) family, serving pivotal functions in cellular adhesion, proteolytic processes, and signal transduction mechanisms (Zhong and Khalil 2019). Research has demonstrated that genetic polymorphisms within the ADAM33 locus are correlated with an increased susceptibility to bronchial disorders, notably asthma (Deng et al. 2017), and this gene also represents a potential biomarker for inflammation and a viable therapeutic target in individuals afflicted with chronic obstructive pulmonary disease (Fachri et al. 2021). Nevertheless, the specific role of ADAM33 in GERC remains uncharted territory, and the potential influence of THD on ADAM33 expression levels is yet to be elucidated.

Rap guanine nucleotide exchange factor (RAPGEF/Epac), a guanine nucleotide exchange factor directly activated by cyclic adenosine monophosphate (cAMP), regulates cellular responses by activating Rap small GTPases (Lee 2021; Gloerich and Bos 2010). This study investigated the effect of THD on AHR through in vivo and in vitro experiments, and delved into the molecular mechanisms by which THD influenced AHR via the Epac1/Rap1 signaling pathway, proposing novel therapeutic avenues for GERC.

## 2 Materials and Methods

### 2.1 Preparation of THD

Tongjiang Hewei formula was purchased from Shanghai Wanshicheng Pharmaceutical Co. Ltd. (Shanghai, China). The THD was prepared in boiling water at a concentration of 0.44 g/mL.

### 2.2 Construction of a Guinea Pig Model of AHR

Male Hartley guinea pigs (6 weeks old, weighing 350–380 g) were obtained from Beijing Vital River Laboratory Animal Technology Co. Ltd. (Beijing, China) and acclimatized for 1 week. The guinea pigs were divided into the following groups (*n* = 10): Normal group, Sham group (Sham), AHR model group, AHR + LD group (AHR guinea pigs were treated with low‐dose THD, 0.5 mL/day/per guinea pig); AHR + MD group (medium‐dose THD, 1.0 mL/day/per guinea pig); AHR + HD group (high‐dose THD, 1.5 mL/day/per guinea pig), AHR + PD group (positive drugs, Rabeprazole and Mosapride) and AHR + HD + Epac1/Rap1 activator group (high‐dose THD and Epac1/Rap1 activator). The AHR guinea pig model was established by inserting a 5F gastric tube into the lower esophagus. To ensure accurate and consistent placement of the tube tip in the distal esophagus and to prevent any risk of proximal placement or airway exposure, the following measures were taken. First, based on preliminary anatomical measurements in guinea pigs of identical age and weight, the tube was inserted to a pre‐determined length from the incisors. Second, the infusion of hydrochloric acid containing 0.5% pepsin (0.1 mmol/L) was administered slowly at 8 drops/min for 20 min/day. During this period, animals were maintained in an upright position to facilitate the passage of the solution into the stomach via gravity and peristalsis, thereby minimizing the potential for reflux. This procedure was conducted for a total of 14 days (Li et al. 2010). The guinea pigs in the sham group underwent an identical procedure but were infused with normal saline. The Epac1/Rap1 activator, 8‐(4‐chlorophenylthio)‐2′‐O‐methyl‐adenosine 3′,5′‐cyclic monophosphate (8‐CPT, 100 μM) (Sigma‐Aldrich, St. Louis, MO, USA), was injected into the dorsal neck region of guinea pigs in the AHR + HD + Epac1/Rap1 activator group. Subsequently, the guinea pigs were anesthetized through inhalation of a 5% isoflurane‐oxygen mixture and then euthanized to obtain respiratory tissue samples. The trachea was carefully dissected out and cleaned of surrounding connective tissue. For molecular biology analyses, the tracheal tissues were immediately snap‐frozen in liquid nitrogen and stored at −80°C until use.

In this study, all animal experiments were confirmed to the Guide for the Care and Use of Laboratory Animals and were sanctioned by the ethics committee of Yueyang Hospital of Integrative Traditional Chinese and Western Medicine, Shanghai University of Traditional Chinese Medicine (YYLAC‐2024‐234‐2).

### 2.3 Measurement of Lung Resistance (RL)

After anesthesia, guinea pigs were placed in a supine position and secured within the AniRes2005 Animal Lung Function System (BestLab, Beijing, China). The skin was incised to separate the subcutaneous tissue, exposing the trachea which was then connected to a ventilator. An endotracheal tube was inserted, with a respiratory ratio set at 1:1.5, a breathing frequency of 60 times/min, and a tidal volume of 6 mL/kg. The external jugular vein was isolated and cannulated, with the needle handle fixed and the chamber sealed. Acetylcholine diluted with saline (0, 3.125, 6.25, 12.5, 25, 50 μg/kg) was injected intravenously in a stepwise manner, and each dose was injected intravenously in a stepwise manner. The RL (cm H_2_O·mL^−1^·s) was continuously measured and recorded by the system's software. The next dose was administered after the RL value returned to normal levels. The peak RL value after each acetylcholine challenge was used to plot the dose–response curve.

### 2.4 Preparation of Drug‐Containing Serum

Male guinea pigs (6–8 weeks old, weighing 350–380 g) were randomly divided into the following groups: Normal, Sham, AHR, AHR + HD, AHR + PD, and AHR + HD + Epac1/Rap1 activator groups (*n* = 10). Gastric gavage intervention was performed on each medicated group, with a volume of 10 mL/kg, once daily for 7 consecutive days. Two hours after the last administration, the guinea pigs were anesthetized, and whole blood was collected from the abdominal aorta, followed by centrifugation to separate the serum.

### 2.5 Hematoxylin–Eosin (HE) Staining

Tissue sections were successively immersed in xylene and gradient ethanol. The sections were stained with hematoxylin for 2 min, and then rinsed. Hydrochloric acid ethanol was added for differentiation, followed by rinsing. Subsequently, the sections were stained with an eosin staining solution. The sections were then dehydrated by successive immersion in gradient ethanol and xylene. Finally, the sections were mounted with neutral balsam, observed under a microscope, and photographed.

### 2.6 Immunohistochemistry (IHC)

Paraffin‐embedded sections were dewaxed and rehydrated by immersion in xylene and gradient ethanol, then immersed in 0.01 M pH 6.0 citrate buffer. Endogenous peroxidase‐blocking solution and goat serum‐blocking solution were applied successively. The sections were incubated with smooth muscle‐myosin heavy chain (sm‐MHC) and alpha‐smooth muscle actin (α‐SMA/α‐actin) primary antibodies (1/400, Abcam, Cambridge, USA) overnight at 4°C. Subsequently, they were incubated with secondary antibodies (1/200, Abcam) for 30 min. The sections were then stained with Diaminobenzidine and hematoxylin. Finally, the sections were mounted with neutral balsam and observed under a microscope.

### 2.7 Transcriptome Sequencing Analysis

RNA samples were extracted using TRIzol from tissue samples of the AHR and AHR + HD groups for transcriptome sequencing. The quality and integrity of RNA samples were evaluated using the Nano‐100 (Allsheng, Hangzhou, China), after which sequencing libraries were prepared. Transcriptomic profiling was subsequently performed on the Illumina NovaSeq 6000 sequencing platform (Illumina, San Diego, California, USA). Gene differential expression analysis was performed using DESeq2. Differentially expressed genes (DEGs) were filtered based on |log_2_FoldChange| ≥ 1.5 and *p*‐value < 0.05. Gene Ontology (GO) and Kyoto Encyclopedia of Genes and Genomes (KEGG) enrichment analyses were performed.

### 2.8 Real‐Time Fluorescence Quantitative Polymerase Chain Reaction (RT‐qPCR)

Total RNA was isolated from each cellular and tissue group utilizing the TRIzol reagent. The RNA concentration was ascertained on a Nano‐100 instrument (Allsheng). Subsequently, cDNA synthesis was executed using the SweScript All‐in‐One First‐Strand cDNA Synthesis Supermix for qPCR kit (Servicebio, Wuhan, China). RT‐qPCR analysis of the cDNA samples was carried out with SYBR Premix EX Taq (Takara, Tokyo, Japan) under specified conditions: initial denaturation at 95°C for 30 s, followed by 40 cycles of denaturation at 95°C for 10 s and annealing at 60°C for 30 s. GAPDH served as the internal control. The Ct values obtained were scrutinized using the 2^−ΔΔCt^ method, and the experiments were replicated thrice. Primer sequences were detailed in Table S1.

### 2.9 Western Blotting

Cellular and tissue samples underwent lysis via RIPA to procure total protein specimens. Quantification of protein concentration was executed utilizing the Pierce BCA Protein Assay Kit (Thermo Fisher Scientific). Proteins were segregated on a 10% sodium dodecyl sulfate–polyacrylamide gel electrophoresis, and subsequently transferred onto a polyvinylidene fluoride membrane. Following a blockade in 5% non‐fat milk, an overnight incubation at 4°C with primary antibodies (1/1000) ensued, including antibodies against ADAM33 (PA5‐28128, Thermo Fisher Scientific), RhoA (ab187027, Abcam), myosin phosphatase (MYPT1, ab59235, Abcam), p‐MYPT1 (ab59203, Abcam), serum response factor (SRF, ab53147, Abcam), sm‐MHC (ab133567, Abcam), α‐actin (ab5694, Abcam), Epac1 (ab109415, Abcam), Rap1 (ab272863, Abcam), and GAPDH (ab9485, Abcam). Thereafter, a one‐hour incubation at room temperature with corresponding goat anti‐rabbit secondary antibodies (1/5000, Abcam) transpired. Each experimental iteration was replicated thrice. Upon the incubation plate of the Tanon 5200 chemiluminescence imaging system (Tanon, Shanghai, China), the development of the membrane was conducted through enhanced chemiluminescence solution, culminating in the exposure and capture of images.

### 2.10 Cell Culture

To conduct the experiment, guinea pigs from both the normal and model groups were anesthetized, and their lung tissues along with tracheobronchial structures were isolated. Subsequently, the guinea pigs were euthanized. Under a super‐clean bench, the bronchial trees were separated, minced using ophthalmic scissors (to a size of ≤ 1 mm), digested, centrifuged, and then transferred into 25 mL culture flasks where the tissue blocks were evenly spread across the bottom. These were placed in an incubator for semi‐open culture, and the medium was changed after 3 days. Once the cells reached confluence, they were purified using a differential adhesion technique to obtain a higher purity of airway smooth muscle cells (ASMCs), which were then sub‐cultured.

The third‐generation ASMCs were seeded into a 96‐well plate for further cultivation, and divided into the following groups: Control; AHR; AHR + HD; AHR + HD + ROCK inhibitor; AHR + HD + oe‐ADAM33; AHR + HD + si‐ADAM33; AHR + HD + Epac1/Rap1 activator. The Control and AHR group cells were treated with normal serum, while the other groups were treated with serum containing respective drugs for 24 h. ASMCs were treated with Epac1/Rap1 activator (8‐pCPT, 100 μM) for 6 h. To verify the role of ADAM33, siRNA targeting ADAM33 (si‐ADAM33‐1/2/3), as well as corresponding negative controls (si‐NC), were synthesized by Ribobio Company (Guangzhou, China). The coding sequence of ADAM33 was cloned into the pcDNA3.1 vector plasmid (oe‐ADAM33), with the empty vector plasmid (oe‐NC) serving as the control group. Transfection of ASMCs in each group was performed using Lipofectamine 3000 (Thermo Fisher Scientific) for 48 h. Additionally, ROCK inhibitor treatment (Y‐27632, 10 μmol/L, Sigma‐Aldrich) was applied to the ASMCs to investigate the role of the RhoA/ROCK pathway.

### 2.11 Data Analysis

All data were processed using GraphPad Prism 7.0 statistical software. Continuous variables were presented as mean ± standard deviation. Comparisons between two groups were performed using a *t*‐test (Li et al. 2010), while comparisons among multiple groups were conducted using One‐Way Analysis of Variance (ANOVA). Post‐ANOVA pairwise comparisons were carried out using Tukey's multiple comparisons test. The 95% confidence intervals for the median differences were provided in Table S2. A *p*‐value of < 0.05 was considered to indicate statistically significant differences.

## 3 Results

### 3.1 THD Improves GERC Airway Hyperresponsiveness in AHR Guinea Pigs

**FIGURE 1:**
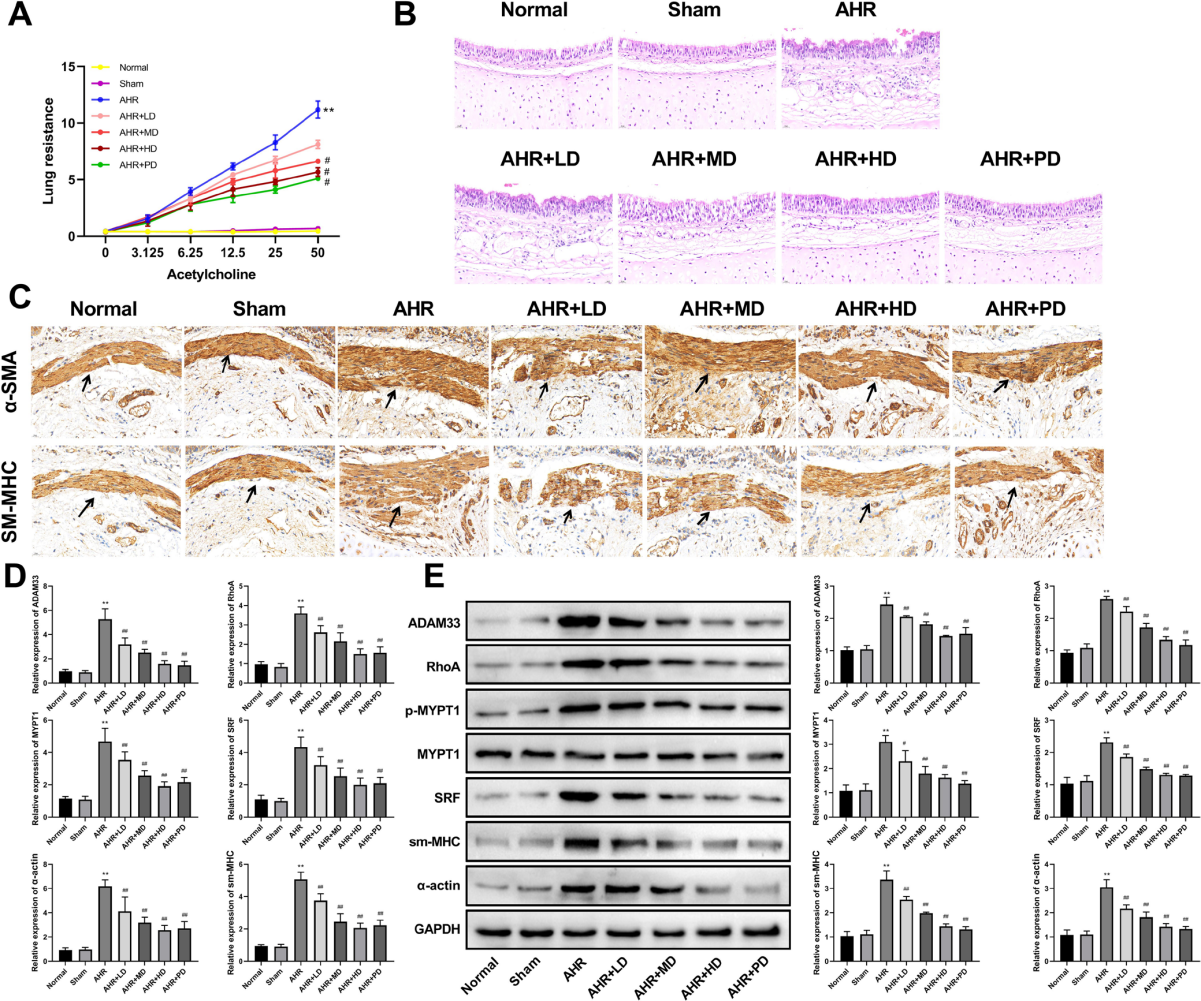
THD ameliorated airway hyperresponsiveness (AHR) in guinea pigs with AHR. (A) Airway resistance measurement. (B) Hematoxylin–Eosin staining of lung tissues (scale bar: 20 μm, Magnification: 400×). (C) Immunohistochemical staining for alpha‐Smooth Muscle Actin (α‐SMA/α‐Actin) and smooth muscle Myosin Heavy Chain (sm‐MHC) (scale bar: 20 μm, Magnification: 400×). (D) Real‐time fluorescence quantitative polymerase chain reaction (RT‐qPCR) was performed to measure the levels of a disintegrin and metalloprotease domain 33 (ADAM33), RhoA, myosin phosphatase (MYPT1), Serum Response Factor (SRF), sm‐MHC, and α‐Actin. (E) Western blotting analysis for ADAM33, RhoA, p‐MYPT1, MYPT1, SRF, sm‐MHC, and α‐Actin expression. ^**^ *p* < 0.01 versus Normal group, ^#^ *p* < 0.05, ^##^ *p* < 0.01 versus AHR group.

To investigate the effects of THD on GERC, we established an AHR guinea pig model and examined the effects of low, medium, and high doses of THD. The results from RL measurements showed that, compared to the Normal group, there was no significant difference in the Sham group, while the RL curve in the AHR group was significantly elevated. Compared to the AHR group, the RL curves in the AHR + LD, AHR + MD, AHR + HD, and AHR + PD groups were significantly reduced (Figure 1A).

The histological examination via HE staining elucidated that in both the Normal and Sham cohorts, there was an absence of notable inflammatory cell infiltration in the vicinity of the trachea, the pulmonary parenchyma remained unblemished, and the alveolar septa were devoid of congestion and edema. Conversely, in the AHR group, a proliferation of inflammatory cells was conspicuous around the trachea, the structural integrity of lung tissue was compromised, the bronchial wall thickness was augmented, and an abundance of mucus secretion was evident within the bronchial lumen. When juxtaposed with the AHR group, the AHR + LD, AHR + MD, AHR + HD, and AHR + PD groups exhibited a diminished infiltration of inflammatory cells to varying extents (Figure 1B).

IHC revealed that relative to the Normal group, α‐SMA and sm‐MHC positive cells in the Sham group displayed no significant deviation, whereas a marked elevation in their expression was observed in the AHR group. In comparison to the AHR group, the α‐SMA and sm‐MHC positive cells in the AHR + LD, AHR + MD, AHR + HD, and AHR + PD groups experienced a significant reduction (Figure 1C).

Furthermore, RT‐qPCR and western blot assays, which scrutinized the expression of ADAM33, RhoA, MYPT1, SRF, α‐actin, and sm‐MHC in the airway smooth muscle of guinea pigs across the various groups, disclosed that in relation to the Normal group, the Sham group manifested no substantial difference in the expression of these proteins. However, a pronounced augmentation in their expression was discerned in the AHR group. When contrasted with the AHR group, the expression of ADAM33, RhoA, MYPT1, SRF, α‐actin, and sm‐MHC in the AHR + LD, AHR + MD, AHR + HD, and AHR + PD groups underwent a significant diminution (Figure 1D,E).

### 3.2 THD Inhibits the Contraction of ASMCs via Suppressing ADAM33

**FIGURE 2:**
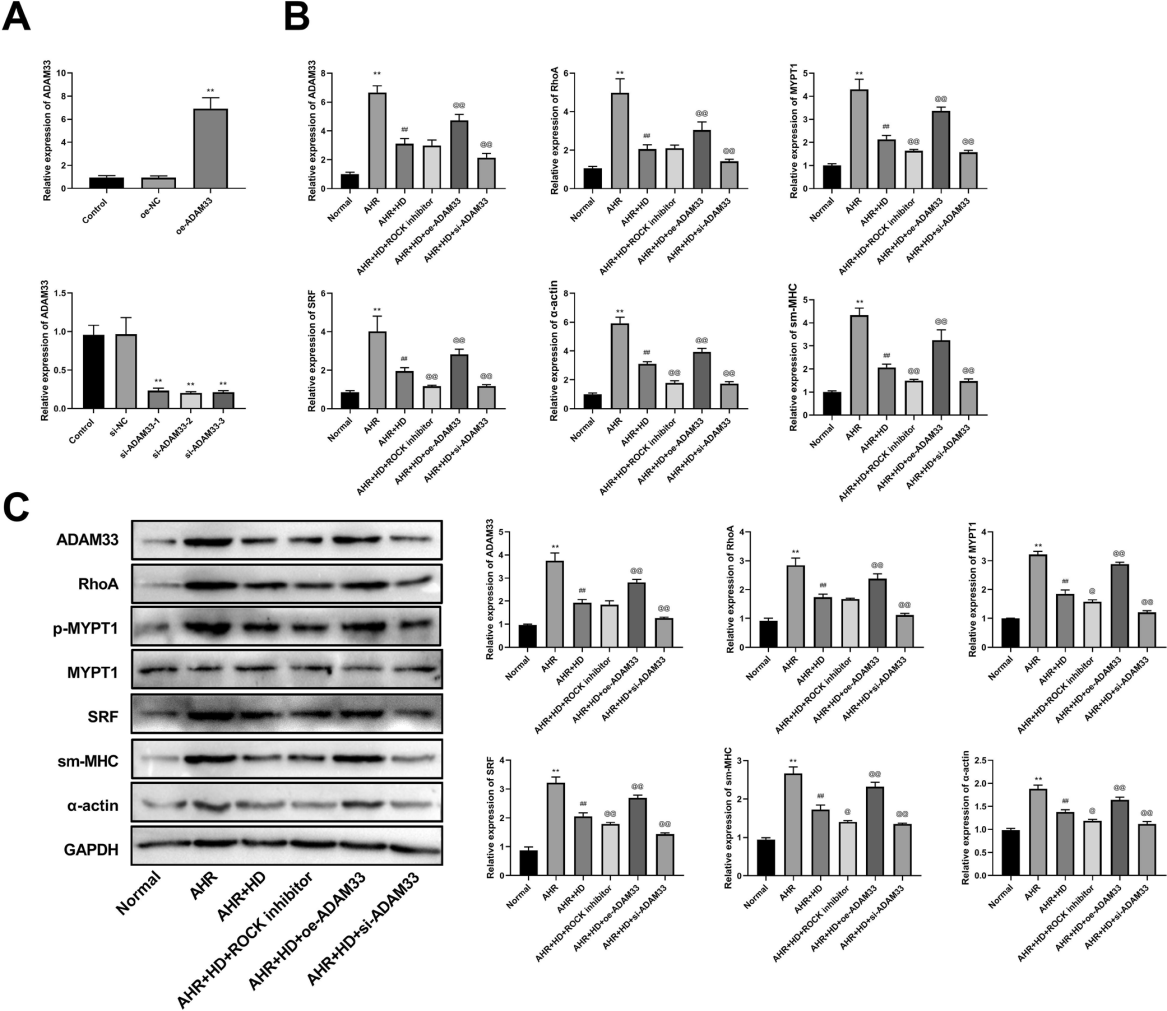
THD inhibited airway smooth muscle cell contraction. (A) RT‐qPCR detection for ADAM33 expression. (B) RT‐qPCR detection for ADAM33, MYPT1, SRF, sm‐MHC and, α‐Actin levels. (C) Western blotting detection for ADAM33, RhoA, p‐MYPT1, MYPT1, SRF, sm‐MHC, and α‐Actin expression. ^**^ *p* < 0.01 versus Normal group, ^##^ *p* < 0.01 versus AHR group, ^@^ *p* < 0.05, ^@@^ *p* < 0.01 versus AHR + HD group.

The ASMCs were classified into several groups, namely: the Control group, the AHR group, the AHR + HD group, the AHR + HD + ROCK inhibitor group, the AHR + HD + oe‐ADAM33 group, and the AHR + HD + si‐ADAM33 group. The RT‐qPCR experimental results demonstrated that, in comparison with the Control group, there existed no significant disparity in ADAM33 expression between the oe‐NC and si‐NC groups. Compared with the oe‐NC group, the ADAM33 expression in the oe‐ADAM33 group exhibited a significant augmentation. Compared with the si‐NC group, the ADAM33 expression in the si‐ADAM33 group displayed a remarkable diminution (Figure 2A). Moreover, RT‐qPCR outcomes, as well as the western blot, revealed that relative to the Control group, the expression of ADAM33, RhoA, MYPT1, SRF, α‐actin, and sm‐MHC in the AHR group significantly escalated. In contrast to the AHR group, the expression of these genes in the AHR + HD group notably declined. Compared with the AHR + HD group, the expression of MYPT1, SRF, α‐actin, and sm‐MHC in the AHR + HD + ROCK inhibitor group underwent a significant reduction. Conversely, in the AHR + HD + oe‐ADAM33 group, the expression of ADAM33, RhoA, MYPT1, SRF, α‐actin, and sm‐MHC substantially increased, while in the AHR + HD + si‐ADAM33 group, their expression significantly diminished (Figure 2B,C).

### 3.3 Transcriptome Sequencing Analysis

A total of 390 up‐regulated and 981 down‐regulated DEGs were screened in both the AHR group and the AHR + HD group, with the top 10 up‐regulated and down‐regulated DEGs listed in Table S3. A volcano plot of the DEGs was generated (Figure S1A). To visually reflect the changes in the DEGs, hierarchical clustering analysis of these genes along with the samples from both groups was conducted using R software, resulting in a heatmap of the DEGs (Figure S1B). Several DEGs, including PAQR7, CPLX3, SLURP2, MMP13, SPA17, and NR4A1, were selected for validation by RT‐qPCR, which found that compared to the AHR group, PAQR7, CPLX3, and SLURP2 were significantly up‐regulated, while MMP13, SPA17, and NR4A1 were significantly down‐regulated in the AHR + HD group (Figure S1C), which was consistent with the transcriptome sequencing results.

To elucidate the functional implications of DEGs, GO and KEGG pathway analyses were performed. The GO enrichment analysis results for the DEGs were categorized into three main groups: Biological Processes, Cellular Components, and Molecular Functions. The top 30 GO terms with the smallest *p*‐values, indicating the most significant enrichment, were selected from each category, such as cilium, cell projection and axoneme in Cellular Components; tubulin binding, binding and nucleoside diphosphate kinase activity in Molecular Functions; cilium organization, cilium assembly and plasma membrane‐bounded cell projection assembly in Biological Processes (Figure S1D). Based on the KEGG pathway enrichment analysis of the DEGs, the top 30 pathways with the lowest *p*‐values, representing the most significantly enriched pathways, demonstrated that THD exerted its effects through signaling pathways involving P53, Rap1, AMPK, and others (Figure S1E).

### 3.4 THD Inhibits the Epac1/Rap1 Pathway in ASMCs

**FIGURE 3:**
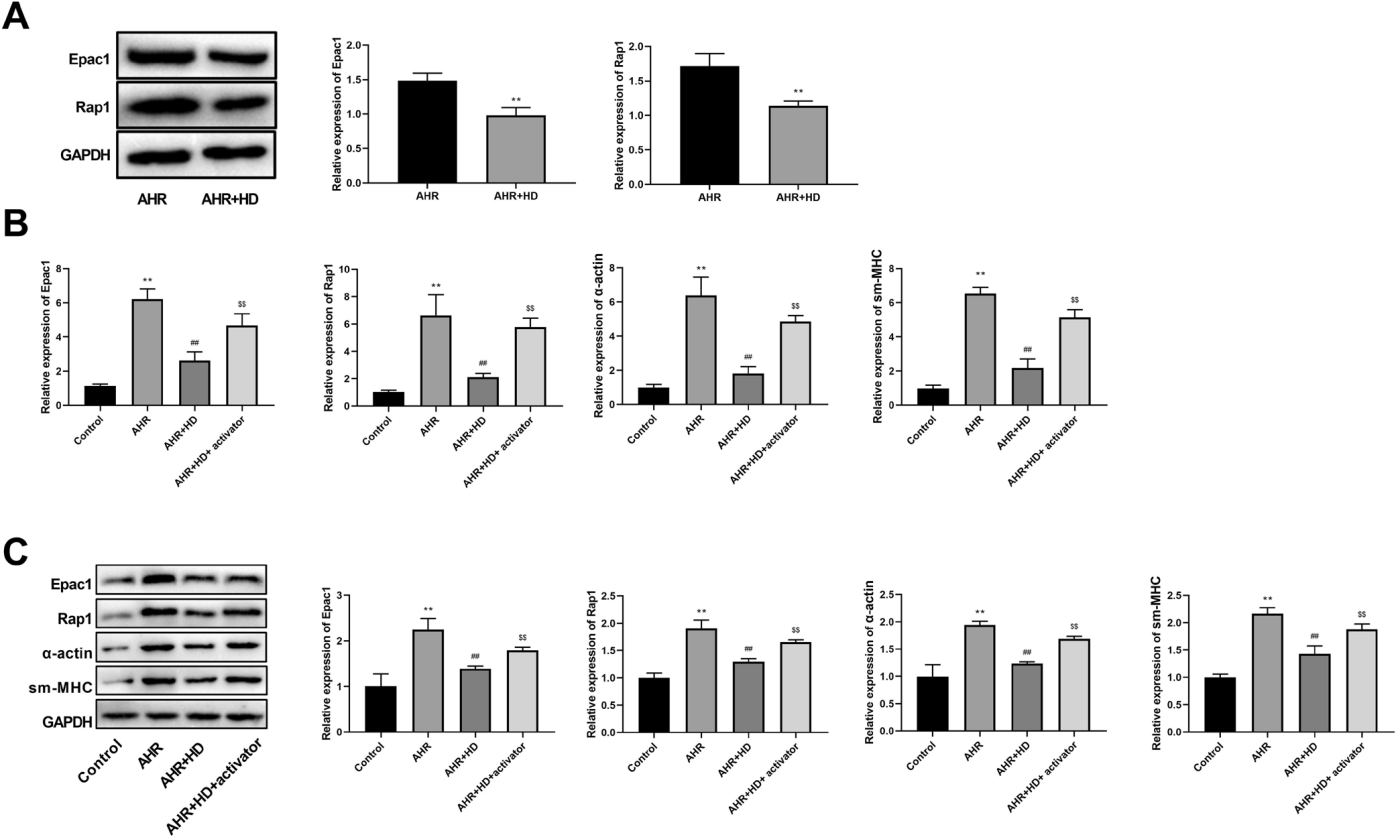
THD inhibited the Epac1/Rap1 pathway in airway smooth muscle cells. (A) Western blotting was performed to detect protein expression. **p < 0.01 versus AHR group. (B) RT‐qPCR assessment of Epac1, Rap1, sm‐MHC, and α‐Actin levels. (C) Western blotting detection of Epac1, Rap1, sm‐MHC, and α‐Actin protein levels. ^**^ *p* < 0.01 versus Control group, ^##^ *p* < 0.01 versus AHR group, ^$$^ *p* < 0.01 versus AHR + HD group.

Western blot analysis was used to validate the expression of proteins related to the Rap1 pathway, which indicated that compared to the AHR group, the expression of Epac1 and Rap1 proteins was significantly reduced in the AHR + HD group (Figure 3A). To investigate the impact of THD on the Epac1/Rap1 pathway in vitro, the expression levels of Epac1, Rap1, α‐actin, and sm‐MHC were measured by RT‐qPCR and western blot, and the Epac1/Rap1 activator was employed. Compared to the Control group, the expression of Epac1, Rap1, α‐actin, and sm‐MHC significantly increased in the AHR group. In comparison to the AHR group, the expression of Epac1, Rap1, α‐actin, and sm‐MHC was significantly suppressed in the AHR + HD group. Furthermore, the Epac1/Rap1 activator significantly reversed the expression of Epac1, Rap1, α‐actin, and sm‐MHC compared to the AHR + HD group (Figure 3B,C).

### 3.5 THD Improves AHR in GERC by Inhibiting the Epac1/Rap1 Pathway

**FIGURE 4:**
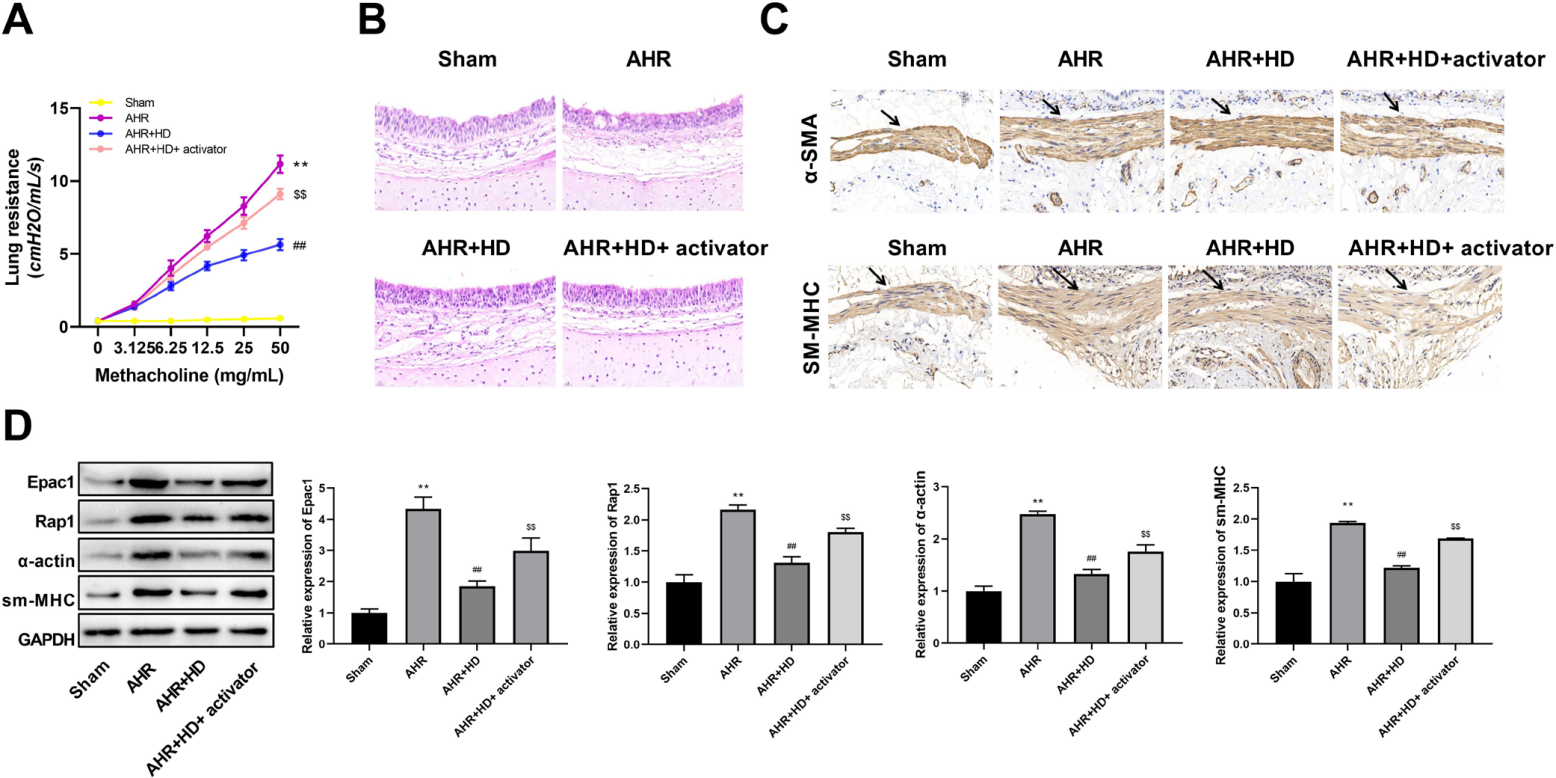
THD improved GERC‐induced AHR via suppressing the Epac1/Rap1 pathway. (A) Pulmonary resistance measurement. (B) Hematoxylin–Eosin staining of lung tissues (scale bar: 20 μm, magnification: 400×). (C) Immunohistochemical staining for α‐SMA and sm‐MHC (scale bar: 20 μm, magnification: 400×). (D) Western blotting analysis demonstrating changes in protein expression along the Epac1/Rap1 pathway. ^**^ *p* < 0.01 versus Sham group, ^##^ *p* < 0.01 versus AHR group, ^$$^ *p* < 0.01 versus AHR + HD group.

To determine the specific mechanism by which the THD improved AHR in vivo, a feedback experiment using an Epac1/Rap1 activator was conducted. Airway resistance was assessed, revealing that the RL variation curve was notably elevated in the AHR group when compared with the Sham group. Conversely, this curve exhibited a significant decline in the AHR + HD group relative to the AHR group. Furthermore, in comparison to the AHR + HD group, the RL variation curve displayed a conspicuous increase in the AHR + HD + Epac1/Rap1 activator group (Figure 4A).

Subsequently, respiratory tissue samples from the guinea pigs were obtained for further analysis. HE staining revealed that in the Sham group, there was an absence of significant inflammatory cell infiltration surrounding the trachea, and the lung tissue structure remained intact without congestion or edema. Conversely, in the AHR group, extensive inflammatory cell infiltration was observed around the trachea, accompanied by structural damage to the lung tissue, thickened tube walls, and a substantial amount of mucus secretion within the bronchial lumen. Compared to the AHR group, THD significantly ameliorated the phenomenon of tracheal inflammatory cell infiltration in the AHR + HD group. However, in contrast to the AHR + HD group, the AHR + HD + Epac1/Rap1 activator group exhibited a significant exacerbation of tracheal inflammatory cell infiltration (Figure 4B).

IHC was employed to assess the expression levels of α‐SMA and sm‐MHC proteins within airway smooth muscle, which demonstrated that, relative to the Sham group, the expression of both α‐SMA and sm‐MHC was significantly upregulated in the AHR group. Conversely, when compared to the AHR group, these proteins exhibited a significant downregulation in the AHR + HD group. In contrast, compared to the AHR + HD group, the expression of α‐SMA and sm‐MHC was notably uplifted in the AHR + HD + Epac1/Rap1 activator group (Figure 4C).

Western blot analysis was utilized to quantify the expression of pivotal proteins within the Epac1/Rap1 pathway, alongside α‐actin and sm‐MHC, in airway smooth muscle, revealing that, relative to the Sham group, the expression of these proteins was significantly elevated in the AHR group. Conversely, when compared with the AHR group, their expression experienced a significant decline in the AHR + HD group. In contrast, in comparison with the AHR + HD group, the expression of these proteins witnessed a notable increase in the AHR + HD + Epac1/Rap1 activator group (Figure 4D).

## 4 Discussion

GERC is a complex disorder characterized primarily by chronic cough, which impairs patients' quality of life (Ahmad and Iyer 2022). In recent years, numerous studies have demonstrated that TCM exhibited good therapeutic effects in the treatment of GERD and GERC, providing new clinical treatment options (Xu et al. 2022; Lin, Huang, et al. 2020). This study revealed that THD markedly attenuated AHR, as evidenced by integrated in vivo and in vitro analyses. Mechanistic investigations identified dual inhibitory effects of THD on ADAM33 expression and RhoA/ROCK pathway activation, effectively blunting airway smooth muscle contractility. Transcriptomic profiling further implicated Epac1/Rap1 signaling as a contributory axis in THD's anti‐AHR activity. Crucially, pharmacological activation of Rap1 in vivo abolished THD‐mediated AHR improvement, directly validating the pathway's mechanistic indispensability.

GERC is attributed to vagal nerve stimulation and microaspiration of refluxate into the tracheobronchial tree (Chen et al. 2018). Direct contact of acidic refluxate with the airways induces inflammation, mucosal damage, and activation of neural reflexes, thereby increasing airway sensitivity and triggering AHR (Chen et al. 2018, 2022). TCM has long been employed as an adjunctive or alternative therapeutic approach for managing GERD and its associated symptoms (Chen et al. 2023). Various herbal constituents and decoctions have demonstrated promising efficacy in alleviating GERD‐related symptoms and modulating airway hyperreactivity. For instance, berberine has been shown to improve pulmonary function in AHR‐afflicted guinea pigs while protecting against tissue damage in the lungs, esophagus, and trachea, suggesting its potential therapeutic value in GERC‐induced AHR (Zou et al. 2022). Zisuzi Decoction ameliorates AHR and partially reverses airway remodeling through inhibition of PI3K/AKT1/mTOR, JAK2/STAT3, and HIF‐1α/NF‐κB signaling pathways, demonstrating effectiveness in cough‐variant asthma management (Nguyen et al. 2023). Shegan Mahuang Decoction attenuates asthmatic AHR by impeding Th2/Th17 differentiation and upregulating CD4 + FoxP3+ regulatory T cells (Lin, Wang, et al. 2020). In this study, THD significantly reduced RL in guinea pigs with AHR and attenuated inflammatory infiltration in the trachea. Additionally, THD inhibited the expression of MYPT1, SRF, α‐actin, and sm‐MHC proteins both in vivo and in vitro, suggesting that THD can alleviate AHR induced by GERC.

Furthermore, ADAM33 has been implicated in the remodeling of airway tissues. Research has reported that ADAM33 is linked with alterations in airway structure among asthma patients, resulting in heightened responsiveness, constriction of the airways, and ultimately diminished therapeutic efficacy (Vishweswaraiah et al. 2023). In an experimental model of allergic asthma utilizing rats, the silencing of ADAM33 has been proven to decrease the proliferation of ASMCs while concurrently enhancing apoptosis (Zhou et al. 2019). Moreover, the knockdown of ADAM33 impedes the migration of human ASMCs through the modulation of the PI3K/AKT/mTOR signaling pathway and regulates the secretion of pertinent cytokines involved in airway vascular remodeling (Yan et al. 2022). Duan et al. discover that overexpression of soluble ADAM33 promotes the hypercontractile phenotype of rat ASMCs and significantly enhances the activation of the Rho/ROCK pathway, leading to AHR in asthma (Duan et al. 2016). Similarly, in vivo and in vitro experiments have shown that overexpressing ADAM33 attenuates the inhibitory effect of THD on AHR‐related proteins and RhoA expression, indicating that THD may alleviate AHR by inhibiting the ADAM33/RhoA/ROCK axis.

Transcriptomic analysis further elucidated the molecular mechanisms underlying THD's modulation of AHR, identifying a critical role of the Epac1/Rap1 signaling pathway. Epac1, activated by cAMP, subsequently activates Rap1 GTPase to mediate diverse cellular processes (Baameur et al. 2016). cAMP modulates the fast sodium current in murine ASMCs via Epac‐mediated signaling pathways, thereby orchestrating bronchodilation and airway remodeling processes (Matthews et al. 2024). Prostacyclin administration demonstrates therapeutic efficacy in ameliorating lipopolysaccharide‐induced acute lung injury and facilitates endothelial barrier restoration through Rap1‐dependent mechanisms (Birukova et al. 2015). Studies demonstrate that Epac exerts protective effects against asthmatic airway inflammation and remodeling through partial suppression of store‐operated Ca^2+^ entry in ASMCs (Chen et al. 2019). In AHR guinea pig models, THD significantly suppressed Epac1/Rap1 signaling, while Rap1 activators markedly attenuated THD's therapeutic effects on AHR, demonstrating that THD alleviated GERC‐induced AHR via the inhibition of Epac1/Rap1 pathway inhibition, and the Epac1/Rap1 pathway has an instrumental part in the therapeutic effect of THD on AHR.

Our study elucidated the dual inhibitory effects of THD on the ADAM33/RhoA/ROCK and Epac1/Rap1 axes, providing a molecular basis for its efficacy against GERC‐induced AHR. These findings position THD as a compelling multi‐targeted therapeutic candidate, particularly in addressing the unmet needs in current Western management of GERD/GERC. A significant proportion of patients respond inadequately to PPIs, which primarily target acid suppression but may not alleviate non‐acid reflux or the ensuing airway complications (Johnston et al. 2016). THD, with its holistic approach rooted in TCM, could serve as a valuable complementary or alternative option for these PPI‐refractory cases, especially when AHR is a prominent feature. When compared to existing therapies, THD presents a distinct advantage profile. Unlike PPIs, which offer symptomatic relief but may not modify the underlying dysmotility or neural hypersensitivity (Wang et al. 2025), THD appears to directly target pathways involved in airway smooth muscle contraction and remodeling. Furthermore, compared to invasive endoscopic procedures or surgery (Kushner et al. 2020), THD is non‐invasive, potentially yielding better patient adherence and a more favorable safety profile.

However, it is also important to acknowledge its limitations, such as the need for individualized prescription by a qualified TCM practitioner and the current lack of large‐scale, randomized controlled trials validating its efficacy and safety against standard Western treatments. In our experimental model, THD administration at various doses did not induce significant adverse effects, suggesting a good tolerability profile. This is consistent with the long‐standing clinical use of its constituent herbs in TCM. Nonetheless, as with any herbal formulation, rigorous quality control to standardize the preparation and continued pharmacovigilance for potential herb‐drug interactions are prudent measures for its future integration into broader clinical practice.

## 5 Conclusions

This study evidenced that THD significantly ameliorated GERC‐induced AHR by inhibiting ADAM33 and RhoA/ROCK signaling pathways, and Rap1 agonist administration reversed the therapeutic effects of THD on AHR. These findings propose a promising therapeutic avenue for enhancing clinical outcomes in patients while offering novel insights into the complex molecular mechanisms underlying both GERD and GERC.

## Author Contributions

Xiulian Zhang and Xueliang Li participated in the project design, project administration, intervention, supervision, writing original draft; data gathering, data analysis, results interpretation, writing review and editing. Yanmei Cheng participated in the project design, supervision, results interpretation, methodology, writing review and editing. Lei Wei and Fangying Liu participated in data analysis, results interpretation, writing review and editing. Li Li participated in data analysis, results interpretation, writing review and editing; Wei Zhang participated in the intervention and methodology. Xiuli Yan participated in the project design, supervision, results interpretation, review and editing. All the authors took part in the experiment. All the authors read and approved the manuscript.

## Ethics Statement

The research was approved by the Ethics Committee of Baoshan Hospital of Shanghai University of Traditional Chinese Medicine (No. YYLAC‐2024‐234‐2). All methods were performed in accordance with the relevant guidelines and regulations.

## Consent

Informed consent was obtained from all subjects.

## Conflicts of Interest

The authors declare no conflicts of interest.

## Supporting information


**Figure S1:** Transcriptomic sequencing analysis. (A) Volcano plot illustrating differentially expressed genes (DEGs). (B) Hierarchical clustering dendrogram based on transcriptomic data. (C) RT‐qPCR validation of expression of hub genes. **p < 0.01 versus AHR group. (D) Gene Ontology analysis of DEGs. (E) Kyoto Encyclopedia of Genes and Genomes analysis of DEGs.

**Table S1:** Primer sequences in this study.

**Table S2:** The 95% confidence intervals for the median differences.

**Table S3:** Top ten up‐ and down‐regulated differentially expressed genes.

## Data Availability

The data that support the findings of this study are available from the corresponding author upon reasonable request.
