# Supplementary figures and images for: Tongjiang Hewei Decoction Improves Airway Hyperresponsiveness in Gastroesophageal Reflux Cough by Inhibiting ADAM33 and Epac1/Rap1 Pathway
Source: Food Sci Nutr. 2025 Dec 18;13(12):e71223. doi: 10.1002/fsn3.71223 (PMC12714587; doi:10.1002/fsn3.71223)

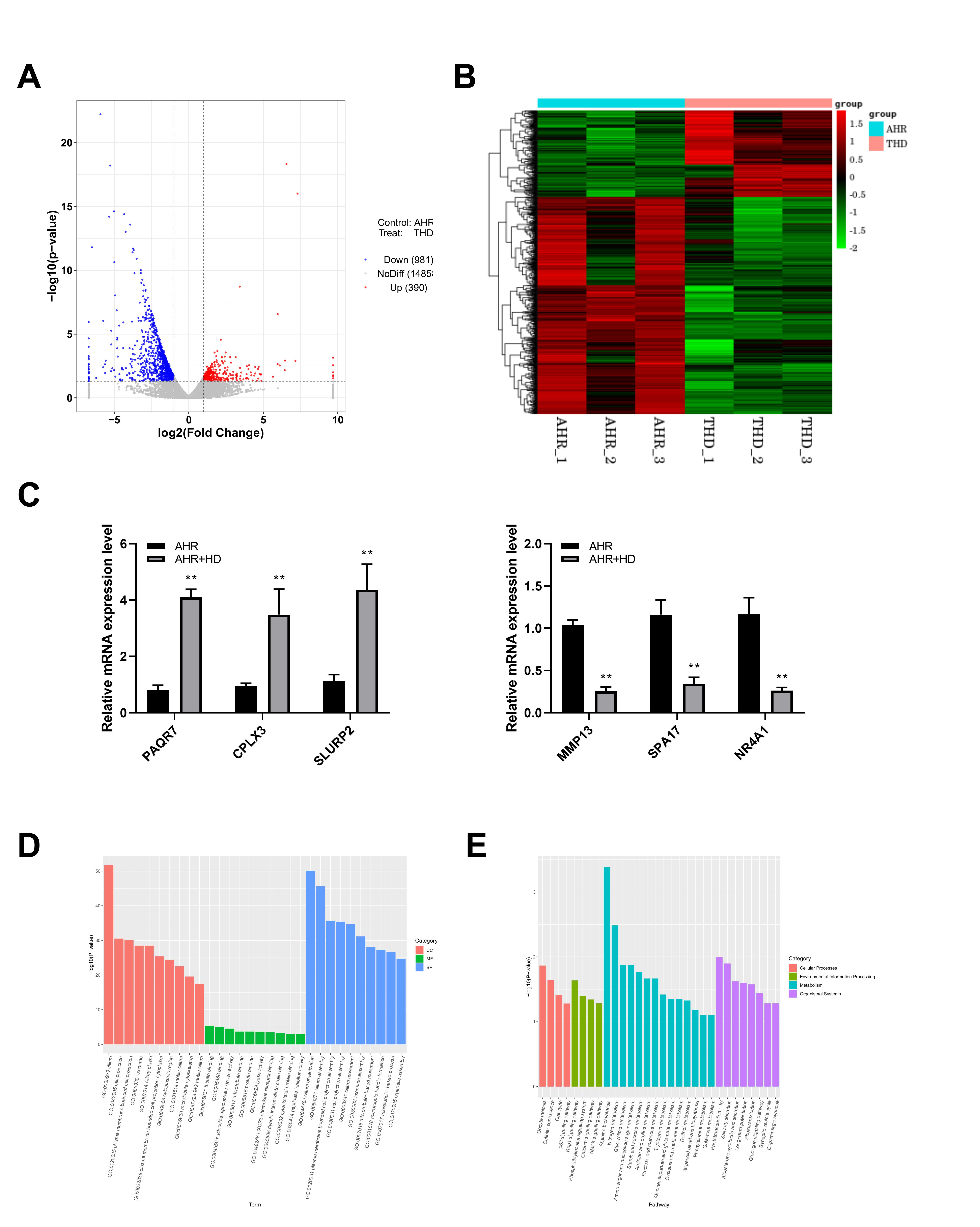

Supplement: Supplementary file 1 — Figure S1: Transcriptomic sequencing analysis. (A) Volcano plot illustrating differentially expressed genes (DEGs). (B) Hierarchical clustering dendrogram based on transcriptomic data. (C) RT‐qPCR validation of expression of hub genes. **p < 0.01 versus AHR group. (D) Gene Ontology analysis of DEGs. (E) Kyoto Encyclopedia of Genes and Genomes analysis of DEGs. [file FSN3-13-e71223-s004.jpg]
