# Supplementary material for: Tongjiang Hewei Decoction Improves Airway Hyperresponsiveness in Gastroesophageal Reflux Cough by Inhibiting ADAM33 and Epac1/Rap1 Pathway
Source: Food Sci Nutr. 2025 Dec 18;13(12):e71223. doi: 10.1002/fsn3.71223 (PMC12714587; doi:10.1002/fsn3.71223)
Supplement: Supplementary file 2 — Table S1: Primer sequences in this study. [file FSN3-13-e71223-s001.docx]

**Supplementary Table 1 Primer sequences in this study**

| **Primer name** | **Sequence (5’-3’)** |
| --- | --- |
| GAPDH-F | GATTTGGCCGTATTGGACGC |
| GAPDH-R | GTGATTCACGCCCATCACGA |
| PAQR7-F | ATGAGGCAGTGAACGTCTGG |
| PAQR7-R | ACATGGCCACACCCACATAG |
| CPLX3-F | CACCACCGCAGCCCCTAAT |
| CPLX3-R | TGGGCAGTCGGTATTTGTCTC |
| SLURP2-F | AGGCTTTGGAGGATGTTCCC |
| SLURP2-R | CCAAGCTGGAGACATCAGGG |
| MMP13-F | GGTTGACAGGCTCCGAGAAA |
| MMP13-R | CAGAGGTGTCACACTGGACC |
| SPA17-F | GGGGCTAAGATAGACGACCG |
| SPA17 -R | GCTGCTTGGATTTTGACAGCA |
| NR4A1 -F | CTTCCAGGTGTACGGTTGCT |
| NR4A1 -R | GTCACAGGTGCATCCAGCAT |
| ADAM33-F | CGCTCATCTTCCAGGTCACA |
| ADAM33-R | GGTCCTGAGGAGCTGGTCTA |
| RhoA-F | CCAAGATGAAACAGGAGCCAGTA |
| RhoA-R | CCAAAGGCGCCAATCCTGT |
| MYPT1-F | GATGCAAGGCAGTGGTTGAA |
| MYPT1-R | GCAAGTGCACCATAACTGCC |
| SRF-F | ACACGACCTTCAGCAAGAGG |
| SRF-R | AAATACCTGTGGGACACCGC |
| α-actin-F | TGGGTATGGGTCAGAAGGACT |
| α-actin-R | GGTGACACCGTCGCCAGA |
| sm-MHC-F | AGCAAGGCTGGATGACGAAA |
| sm-MHC-R | CGCCTCGTTGAGCATGATTG |
| Rap1-F | CGTGGGAAAGTCTGCTCTGA |
| Rap1-R | CGCTCATCTTCCAGGTCACA |
