# Supplementary material for: Tongjiang Hewei Decoction Improves Airway Hyperresponsiveness in Gastroesophageal Reflux Cough by Inhibiting ADAM33 and Epac1/Rap1 Pathway
Source: Food Sci Nutr. 2025 Dec 18;13(12):e71223. doi: 10.1002/fsn3.71223 (PMC12714587; doi:10.1002/fsn3.71223)
Supplement: Supplementary file 4 — Table S3: Top ten up‐ and down‐regulated differentially expressed genes. [file FSN3-13-e71223-s003.docx]

**Supplementary Table 3 Top ten up- and down-regulated differentially expressed genes**

| **Genes** | **Description** | **log2fc** | **pval** | **padj** | **Up/Down** |
| --- | --- | --- | --- | --- | --- |
| MMP13 | matrix metallopeptidase 13 | -5.93 | 5.85E-23 | 9.49E-19 | Down |
| C11orf97 | chromosome 11 open reading frame 97 | -5.02 | 2.41E-15 | 7.81E-12 | Down |
| CDC20B | cell division cycle 20B | -5.35 | 6.26E-15 | 1.45E-11 | Down |
| SFTPD | surfactant protein D | -3.93 | 2.59E-14 | 5.26E-11 | Down |
| PACRG | parkin coregulated | -4.24 | 9.69E-14 | 1.75E-10 | Down |
| SPA17 | sperm autoantigenic protein 17 | -3.70 | 2.60E-12 | 3.51E-09 | Down |
| GLOD5 | glyoxalase domain containing 5 | -3.78 | 3.92E-12 | 4.89E-09 | Down |
| NR4A1 | nuclear receptor subfamily 4 group A member 1 | -3.50 | 1.23E-11 | 1.43E-08 | Down |
| RFK | riboflavin kinase | -3.22 | 9.69E-11 | 9.83E-08 | Down |
| AK8 | adenylate kinase 8 | -3.65 | 1.64E-10 4.71E-19 | 1.48E-07 3.30E-15 | Down |
| PAQR7 | progestin and adipoQ receptor family member 7 | 6.56 | 2.77E-05 | 3.40E-03 | Up |
| HMCN2 | hemicentin 2 | 2.15 | 2.14E-04 | 1.67E-02 | Up |
| PSTPIP2 | proline-serine-threonine phosphatase interactingprotein 2 | 1.92 | 2.77E-04 | 2.04E-02 | Up |
| ABCA6 | ATP binding cassette subfamily A member 6 | 2.47 | 2.81E-04 | 2.05E-02 | Up |
| TINAGL1 | tubulointerstitial nephritis antigen like 1 | 1.73 | 5.36E-04 | 3.22E-02 | Up |
| LYNX1 | Ly6/neurotoxin 1 | 1.84 | 5.81E-04 | 3.39E-02 | Up |
| MSLNL | mesothelin like | 2.42 | 6.32E-04 | 3.63E-02 | Up |
| HK3 | hexokinase 3 | 2.77 | 6.34E-04 | 3.63E-02 | Up |
| CPLX3 | complexin 3 | 2.22 | 6.45E-04 | 3.67E-02 | Up |
| SLURP2 | secreted LY6/PLAUR domain containing 2 | 3.15 | 5.85E-23 | 9.49E-19 | Up |
